# Residual Elbow Instability Treated with a Submuscular Internal Joint Stabilizer: Prospective and Consecutive Series with a Minimum Follow-Up of 12 Months

**DOI:** 10.3390/jcm13226765

**Published:** 2024-11-10

**Authors:** Angelo De Crescenzo, Raffaele Garofalo, Andrea Celli

**Affiliations:** 1 Shoulder and Elbow Unit, Department of Orthopaedic and Traumatology Surgery, Ecclesiastical Entity General Regional Hospital “F. Miulli”, Acquaviva delle Fonti, 70021 Bari, Italy; raffaelegarofalo@gmail.com; 2 Shoulder and Elbow Unit, Department of Orthopaedic Surgery, Hesperia Hospital, 41125 Modena, Italy; celli.andrea.md@gmail.com

**Keywords:** internal joint stabilizer, submuscular, residual instability, surgical approach, elbow, anconeus, IJS

## Abstract

**Background**: The management of residual elbow instability is a challenging and compelling issue for treating physicians. To overcome inherent drawbacks of dynamic external fixators, the internal joint stabilizer (IJS) has been developed, achieving successful results, but it can sometimes cause local tenderness or anesthetic concerns in the subcutaneous layer. In addition, a bulky anconeus can pull the hardware away from the axis of rotation with an increase in the lever arm and potential issues. To address these issues, an alternative approach has been recently described in which the internal device is covered by the anconeus muscle, becoming submuscular, rather than subcutaneous. The aim of this study was to evaluate the effectiveness of this alternative approach to the IJS application in maintaining a concentric elbow during and after device removal in both acute and chronic scenarios. **Methods**: Prospective data collection was performed with consecutive patients who had residual elbow instability treated with an IJS (Skeletal Dynamics, Miami, FL) covered by the anconeus from January 2022 and with a minimum follow-up of 12 months. Results: At a medium follow-up of 16 months, the 16 patients selected had a mean arc of flexion–extension of 123° (range: 0–140°) and a mean pronation-supination arc of 150° (range: 80–80°). The mean MEPS and DASH scores were 90.3 ± 6.2 and 6.3 ± 5.3, respectively. At the last follow-up, elbow stability and concentric reduction were confirmed with radiographic and clinical examinations. **Conclusions**: With a minimum follow-up of 12 months, the present study supports the safety and efficacy of the internal device in a submuscular layer. The clinical outcomes and the rate of recurrent instability are comparable to those achieved with a classic subcutaneous position. Similarly, the complication rate is not affected, and removal surgery is no more aggressive than the classic approach.

## 1. Introduction

Instability of the elbow represents a severe complication that may portend miserable outcomes [1]. In the treatment of an acute elbow injury and, similarly, of a chronic condition, the primary aim for the treating physician is to achieve a stable and congruent joint. For this reason, especially in complex scenarios, it is a common practice to delay joint motion and rehabilitation in order to achieve secondary stability. For example, with a strenuous coronoid fixation or poor soft tissue, elbow immobilization with a cast is usually performed. Nonetheless, early rehabilitation and joint motion have been identified as a crucial component of achieving satisfactory outcomes [2,3]. Thus, primary joint stability must be sought as much as possible to promote early motion and favorable outcomes.

As a matter of fact, persistent or residual instability remains a challenging issue even with advances in surgical techniques. Among the most complex scenario, terrible triad injuries are associated with a rate of recurrent instability ranging from 0% to 33% despite modern treatment protocols [4]. To increase joint stability, supplemental stabilization options can be used either statically or dynamically [5]. The static methods involve rigid immobilization achieved with a static external fixator, a bridging plate, or trans-articular pinning, which provide more stability but delay the rehabilitation [6,7]. Conversely, the dynamic external fixator has been used to provide additional stability and, at the same time, early motion. However, this technique is technically demanding and is associated with a high rate of complications [7,8,9,10].

To address these issues due to intrinsic biomechanical drawbacks, an internal hinge has been introduced [7,11,12,13,14,15,16,17]. The internal joint stabilizer (IJS) was designed by Orbay, who initially crafted an internal hinge with a Steinmann pin [12]. The IJS is less technically demanding to implement and can avoid the biomechanical downsides of an external hinge [7,18]. Since it is an internal stabilizer, its lever arm is reduced, and its axis of rotation is more easily recreated [9,12,19,20]. To date, positive and encouraging results have been reported with a high rate of maintained concentric reduction, coupled with a reasonable range of motion [7,11,12,13,14,15,16,17,18].

Despite a low rate of recurrent instability, the overall complication rate observed may be consistent and may reach up to 65.5% [15,21,22]. As matter of fact, the highest rates have been observed in patients with severe terrible triad injuries [21] and the most frequent complications not directly related to the internal device, such as heterotopic ossification, stiffness, and ulnar neuropathy [7,21]. Conversely, complications due to the IJS are usually less frequent, and they include a radiolucent line around the axial pin and hardware failure [9,17]. These are observed in from 0% to 47% and from 0% to 23% of patients, respectively [7,11,17,21,22]. The highest rate has been described by Sheth et al., who reported four patients with implant disassembling (23%) and eight with radiolucent lines of 1–2 mm (47%) [22].

**Figure 1:**
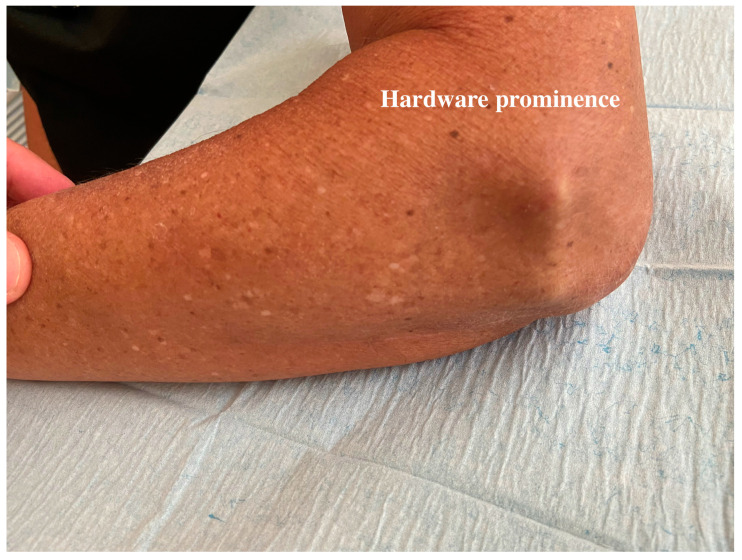
Hardware prominence. The IJS can be remarkably prominent when classically positioned above the anconeus and within a subcutaneous layer, predisposing patients to discomfort and symptomatic hardware.

Being in the subcutaneous layer, the IJS can be prominent and provide some aesthetic and clinical discomfort, especially in skinny patients (Figure 1). In addition, a bulky anconeus could push the IJS away from the lateral epicondyle since the device is positioned on it. To address these issues, a new approach has been recently described that makes the stabilizer submuscular and, accordingly, less clumsy [23]. In this alternative approach, an anconeus muscle flap is developed and used to cover the stabilizer at the end of the procedure. The preliminary results in the first patients have been encouraging [23].

The aim of this manuscript is to describe the clinical and radiographic results of patients treated for residual instability in both acute and chronic scenarios with an IJS covered by the anconeus at a minimum follow-up of 12 months. In addition, specific attention is given to the complication rate observed. The overall outcomes are analyzed and compared with the different series of patients whose results have thus far been published in whom the internal device was used with the classic technique. The hypothesis was that the results would be comparable to those of the classic surgical approach and with potential clinical benefits to the patient.

## 2. Materials and Methods

With the starting date of January 2022, a selection of all patients treated with an IJS (Skeletal Dynamics, Miami, FL, USA) covered by the anconeus for residual elbow instability was performed. The patients, who were prospectively analyzed, were included when an adequate bone stock needed for the internal stabilizer was assessed in both acute and chronic settings (more than 6 weeks from the trauma) and the patients were suitable for treatment with an internal stabilizer. The exclusion criteria were a follow-up of less than 12 months, serious soft tissue injuries, known allergic reactions to chrome cobalt, and infection. The IJS was used in either acute or chronic scenarios. Although the preoperative plan suggested the potential need for additional stabilization, the definitive indication was deemed intraoperatively. In detailed terms, the joint was deemed unstable when a gap was detected on lateral radiographic view within the last 30° of extension.

The surgical technique used has been described in detail in a previous report [23]. Thus, the description of the procedure is not provided again since it was not the aim of the present study.

A radiographic evaluation with both X-rays and a CT scan was routinely performed for each patient, whereas, in the postoperative follow-up, an X-ray was suggested at approximately 2 and 6 weeks and then 3, 6, 12, and 18 months. In this way, the joint reduction maintained and the complications were routinely checked.

At each follow-up visit, a clinical evaluation was performed, seeking any local symptoms and recording the pain with the VAS score (Visual Analogue Score, VAS, rated from 0 to 10), the range of motion (ROM) with a goniometer, and scores like DASH (the Disabilities of the Arm, Shoulder, and Hand score) and the MEPS (Mayo Elbow Performance Score) [24,25].

Removal surgery was not considered a “revision” if it was not conducted for a specific complication related to the IJS [7,11,13,14,15,16,17].

### Statistics

Variables are expressed as medians with interquartile ranges. Wilcoxon’s matched-pairs signed-rank test was used to compare post- and pre-treatment data. All analyses were performed using STATA, version 16 (StataCorp, College Station, TX, USA). A value of *p* < 0.05 was considered significant.

## 3. Results

The selection criteria led to 24 consecutive patients treated with additional stabilization achieved with an IJS covered by the anconeus. Excluding those with less than 12 months of follow-up, the final cohort included 16 patients (11 males and 5 females) with an average age of 45.1 years at presentation (range: 21–60; Table 1, Table 2, Table 3 and Table 4). Five patients had an acute injury (three terrible triad, one simple dislocation, and one anteromedial coronoid fracture with posteromedial rotatory instability, PMRI; Table 1), whereas the remaining 11 cases comprised delayed presentations of acute injuries treated at other institutions (Table 2). The demographic data, injuries, and treatment details are described in Table 1 and Table 2.

At a medium follow-up of 16 months (ranging from 12 to 22 months), the mean arc of flexion–extension was of 123° (range: 0–140°), and the mean pronation–supination arc was of 150° (range: 80–80°; Table 3 and Table 4).

The mean MEPS and DASH score observed were 90.3 ± 6. and 26.3 ± 5.3, respectively (Table 3 and Table 4). The average pain was measured with a VAS score of 1.2 ± 0.4. No patient was lost to follow-up. All the patients were satisfied, without any local discomfort at their last follow-up.

Analyzing the group of patients with chronic elbow disease revealed a significant improvement in the flexion–extension arc (46 ± 15, *p* < 0.001) and both the functional scores (32 ± 13 with *p* < 0.001 for the MEPS and −25 ± 11 with *p* < 0.001 for the DASH score), but not for the pronation–supination arc (20 ± 30, *p* = 0.054).

The second surgery for device removal was performed after a mean of 7.3 months (ranging from 4 to 13) since the first surgery. At the time of the device removal and at the last follow-up, the elbow joint was stable, and concentric reduction was confirmed in radiographic and clinical examinations. In fact, each patient was re-evaluated at least once with both X-rays and clinical examination after device removal. In this way, the follow-up was ensured to be long enough to confirm the maintained joint stability and concentricity.

Complications occurred in 10 patients of the 16 patients (63%), but these did not require additional surgery (Table 3 and Table 4). The radiographic reports showed heterotopic ossifications in five patients (31.3%, 5 out of 16 patients), but these were asymptomatic. Analyzing issues due to the stabilizer revealed that three patients experienced a failure of the hinge in the connecting arm and four a central pin loosening from the connecting arm (43.7% overall, 7 out of 16 patients). However, these implant failures did not lead to any consequences on joint stability, and no patient complained of any symptoms related to the device. For this reason, device removal was planned without any priority, as for the other patients. These complications are not linked to the device position relative to the anconeus but likely to issues with screw tightening. Each patient expressed complete tolerance during the period of treatment with the internal stabilizer, without any complaint or soreness.

## 4. Discussion

The results observed in the current study support the feasibility and safety of the submuscular application, rather than the subcutaneous application, of the IJS, achieved by covering the tool with the anconeus muscle flap. The aim of this new approach is to reduce potential local tenderness and the adverse influence that the anconeus muscle can have on the internal joint stabilizer classically positioned in the subcutaneous layer. With a minimum follow-up of 12 months, the results show no complaints of symptomatic hardware or other adverse events directly related to the different anatomic rapport of the device with the anconeus. The submuscular position does not hinder implant removal. The surgical dissection is no larger, and the anconeus is minimally raised for implant removal.

In either acute or chronic settings for the treatment of elbow injuries, reaching appropriate joint stability is mandatory to prevent detrimental immobilization and poor outcomes [2,3,26]. Static additional stabilization with bridging plates, trans-articular pinning, or static external fixators have been used, but they present obvious drawbacks related to joint violation, issues related to pins, rehabilitation delay, and resultant elbow stiffness [6,7]. Conversely, a dynamic external fixator is characterized by the drawbacks related to the pins, such as nerve injury and fractures, are remarkable being observed in till 67% of the patients [8,9]. In addition, recurrent instability has been observed in up to 30% of cases as a consequence of implant flexibility [7].

Since 2016, the internal stabilizer, developed by Jorge Orbay, has provided the treating physician with an alternative internal stabilizer that is easier to implement and potentially more efficient [7,11,12,13,14,15,16,17]. With a shorter lever arm and easier recreation of the axis of rotation, the internal stabilizer remarkably reduces the biomechanical flaws of hinged external fixation [7]. To date, several studies have shown a high rate of maintained concentric reduction and functional range of motion [7,11,12,13,14,15,16,17]. The single retrospective comparative report between the dynamic external fixator and the IJS by Wynn et al. [27] has shown similar clinical outcomes in the treatment of traumatic acute and chronic elbow instability. However, those authors observed more frequent complications and subsequent procedures in the external fixator group [27]. A recent study reviewed the literature to compare dynamic external fixation with IJS, emphasizing their complication profile [28]. Even though the rates of recurrent instability were not significantly different (4.1% in the IJS group vs. 7.0% in the external fixation group, *p* = 0.11), a dynamic external stabilizer is 1.81 times more likely to be associated with recurrent instability [28]. In addition, rates of device failure were comparable between the groups, but the 14.6% rate of pin-related complications with external fixators is concerning [28].

Even though the severe complications associated with dynamic external fixators are avoided, the overall complication rate is still consistent with an average rate from 21% to 65.5% [15,21,22]. As a matter of fact, most of these complications are not directly related to the internal device. These are represented by ulnar neuropathy, stiffness, and heterotopic ossification [7,21].

Conversely, complications due to the IJS are usually less frequent, and they include a radiolucent line around the axial pin and hardware failure [9,17]. Documenting the highest rate so far reported, Sheth et al. described a 23% and 47% rate of implant disassembling and radiolucent lines, respectively [22].

Compared to an external frame, the IJS is more accepted among patients, especially for complex patients such as those addicted to drugs or tobacco and those with brain injuries or psychiatric diseases [13]. On the other hand, an internal stabilizer requires a second surgery for its removal, and it is potentially symptomatic. In fact, the basal plate, and more frequently the lateral connecting arm, can produce some aesthetic and clinical discomfort (Figure 1). This issue may be more relevant in skinny patients with poor fat tissue; in these patients, wound breakdown represents a real and potential complication after surgical treatment [29]. On the other side of the device, a bulky anconeus muscle, as observed in athletic patients, can drive the connecting arm away from the joint, causing potential constraints to smooth device motion.

To address these potential concerns, a new approach to the IJS application has been recently described [23]. The internal device can be effortlessly covered by an anconeus flap. This surgical approach with an anconeus flap can also afford wider access, helping the surgeon better treat the injury [30,31]. This access can be also achieved easily, completing the anconeus dissection after a Boyd or Kocher approach.

**Figure 2:**
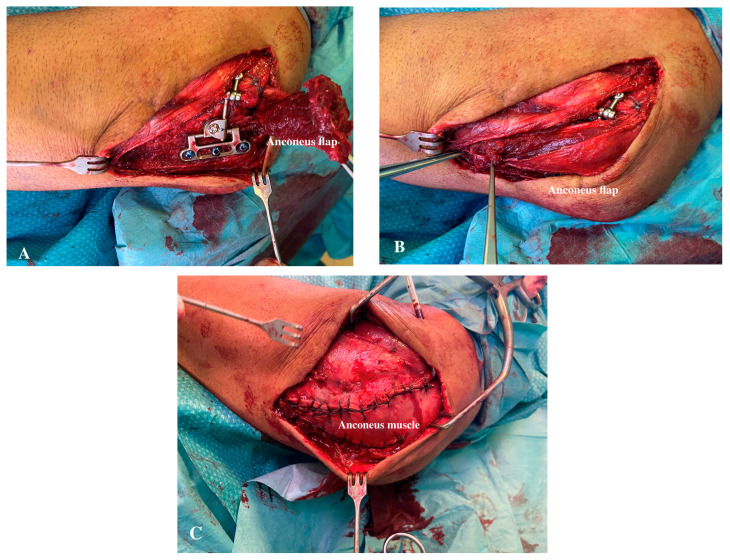
(**A**). Intraoperative clinical IJS evaluation with an anconeus flap. The intraoperative picture shows the definitive IJS implantation with the anconeus flap raised. (**B**). IJS coverage and anconeus flap reattachment. The anconeus flap can, at the end of the procedure, completely cover the internal device. 3 (**C**). Anconeus reattachment. At the end of the procedure, the internal device is not visible being in a submuscular layer.

The results achieved with a minimum follow-up of 12 months have been good and excellent. These are also comparable to those observed in other case series with regard to joint mobility and complications [7,11,12,13,15,16,17,18,21,22]. The internal stabilizer is positioned in a standard fashion, as already described [7,12], but the anconeus is itself to change its position. For this reason, the benefit achieved by the IJS is not interfered. The results are comparable to those of previous reports, supporting our opinion. Even though symptomatic hardware and implant prominence is not a frequent complaint, this approach can reduce or prevent it. As evidenced by this series, a different anconeus position in relation to the IJS does not interfere with the device’s motion and efficacy. With accurate and careful muscle dissection, an anconeus or anconeus–triceps flap can be raised and effortlessly reattached, completely covering the internal hardware at the end of the procedure (Figure 2A–C).

Thus, symptomatic hardware and implant prominence with aesthetic implications can be avoided. In addition, the submuscular position does not increase the potential impingement with bony structures and following pronation–supination limitations, as argued by Sheth et al. [22]. In our opinion, this alternative anconeus position does not interfere with joint motion, as supported by the mean pronation–supination arc observed of 150° (range: 80–80°).

Similarly, the alternative anatomic position of the anconeus does not increase the rate of complications directly due to the internal device. Three patients with failure at the hinge in the connecting arm and four with central pin loosening from the connecting arm (43.7%) have been observed. However, these implant failures did not have any consequences on joint stability, and none of these seven patients complained of any symptoms related to the device. For this reason, device removal was planned without any priority, as for the other patients. These complications are not linked to the device position relative to the anconeus but likely to issues with screws tightening. The patients did not complain of any tenderness or discomfort related to the internal device.

The need for implant removal represents one of the drawbacks of the IJS. Even though suggested by Orbay at 6–8 weeks after index surgery [7], growing evidence of safety and tolerance for the device has been shifting the time of device removal to 3–4 months [13,17]. Some authors advise delaying the removal according to device failure or patient requests [13,22,27,28]. This attitude can be worthwhile when dealing with patients with psychological issues or those who are simply not compliant with the physician’s indications [13]. Nonetheless, the long-term clinical and radiographic outcomes of those patients are still unknown. Accordingly, device removal after at least 3–4 months remains likely the most valuable choice since the potential consequences of a retained device in a long-term follow-up is still unknown.

**Figure 3:**
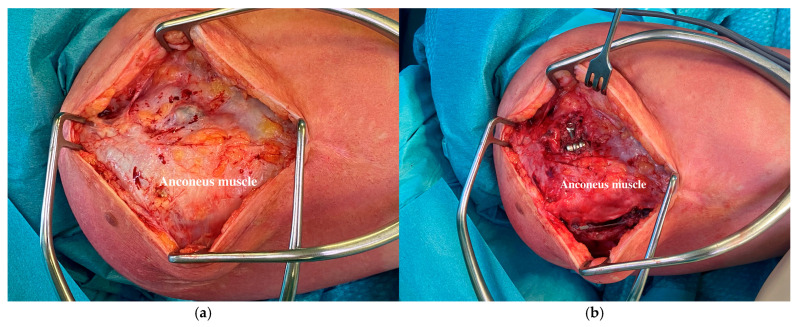
(**a**). Superficial dissection at IJS removal. At the time of device removal, the superficial dissection displays how the IJS is completely covered by the anconeus muscle belly. (**b**). Deep dissection at IJS removal. Two deep windows are created to gain access to the lateral pin (small and lateral window) and the base plate (big and posterior window).

**Figure 4:**
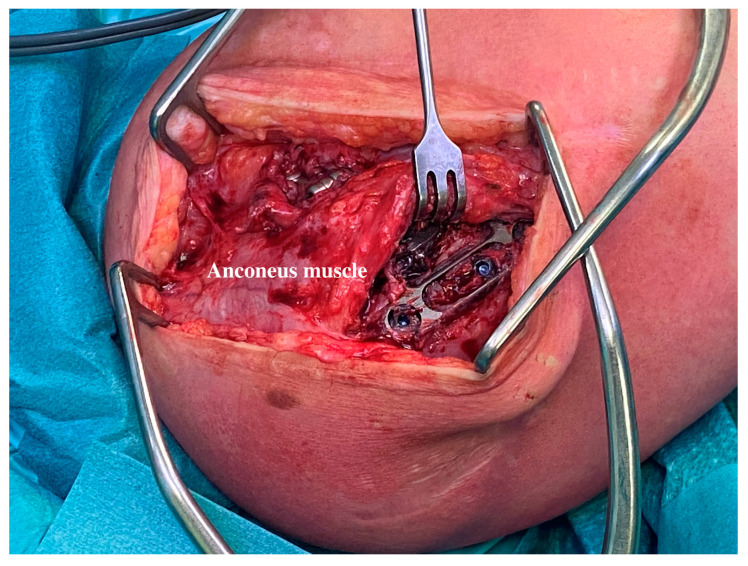
Anconeus flap elevation. The anconeus muscle is minimally raised to expose the base and the distal locking screw.

**Figure 5:**
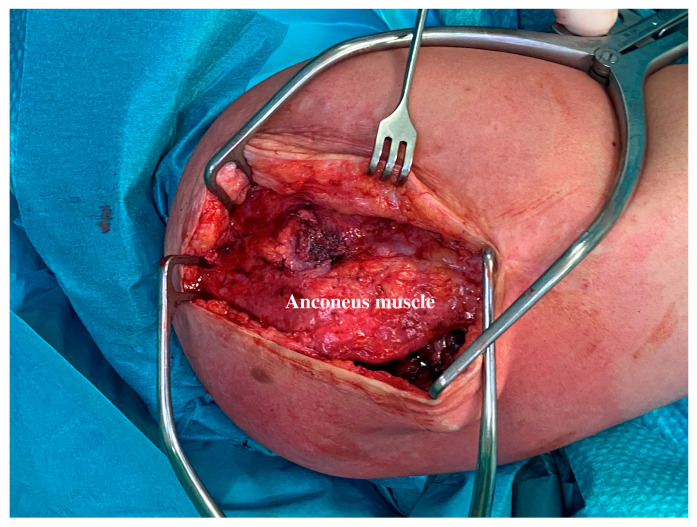
Anconeus muscle condition after IJS removal. The intraoperative picture shows the anconeus tissue quality after the IJJS removal.

At an average of 7.3 months (ranging from 4 to 13), all the patients in the present series underwent device removal. The radiographic evaluation and the intraoperative examination confirmed the stability and concentricity of the joint once the device was removed. The different anconeus position did not involve a more aggressive surgical dissection for the implant removal. The removal procedure can be performed with a single or double skin incision. In any case, two small windows need to be opened around the anconeus (Figure 3a,b). The anconeus is minimally elevated from its ulnar insertion to pull out the base plate and the connecting arm (Figure 4). In the present series, muscle quality was always preserved (Figure 5). This likely happens due to an anastomosed vascular supply that allows for an anconeus detachment from the distal direction to the proximal direction without causing any injury [32,33,34]. Similarly, the neurological supply is normally preserved if the anconeus flap is maintained in continuity with the triceps proximally [23,30,31].

Another potential downside of the internal stabilizer is represented by the interference with the lateral ulnar collateral ligament (LUCL) repair or reconstruction on the humeral side. In fact, the humeral origin of the ligament corresponds to the entry point of the axial pin in the center axis of rotation of the distal humerus. Accordingly, the IJS limits the ability to anatomically repair the LUCL. Thus, this aspect can raise a debate as to what is the best position for ligament reattachment. Placing the anchor anterior and inferior to the isometric point should not restrict the elbow motion [35]. Conversely, a posterior and superior position was preferred for the present cohort to afford a wider area for the tissue to heal. In fact, the humeral footprint of the LUCL is an area, rather than a single point, covering, on average, an area of 26.0 mm^2^ [36]. White and Matullo have recently described a new technique eliminating the footprint competition between LUCL reattachment and IJS humeral pin, and they achieved an isometric placement for both constructs [36]. The technique consists of a LUCL repair using a 4.5 mm SwiveLock anchor (Arthrex) and following axial pin positioning through the hollow center of the suture anchor previously gently drilled with a 2.5 mm drill. This step removes minimal material from the central portion of the suture anchor and does not interfere with the suture of the ligament repair at the bone–anchor interface [36]. The efficacy of this technique relies on an accurate axis of rotation within the distal humerus and a collinearity of the suture anchor and IJS pin [36].

The results observed in the present study could have been skewed by some limitations. As for any single-arm prospective study, the lack of a control group represents a major limitation. Similarly, the sample size and the heterogeneity of the injuries analyzed, either acute or chronic, weakened our conclusion. In the analysis of complications related to the internal device, a CT-study would be worthwhile to better evaluate, for example, the magnitude of bone erosion around the axial pin. However, a radiographic examination is normally used to rule out the presence of radiolucent lines, as in other scenarios, and the clinical evaluation can recognize any related symptoms. Then, a longer follow-up would better assess some complications, like arthrosis, or confirm the consistency of the stability achieved. Device tolerability and the safety of this treatment have been, however, adequately analyzed with a minimum follow-up of 12 months. Further research will be necessary to compare the new technique with the conventional approach.

## 5. Conclusions

When dealing with residual instability in both acute and chronic scenarios, the IJS can be covered by the anconeus muscle. With a minimum follow-up of 12 months, the present study supports the safety and efficacy of the internal device in a submuscular layer. In fact, the clinical outcomes and rate of recurrent instability are comparable to those achieved with a classic subcutaneous position. Moreover, the complication rate is similar, and the removal surgery is no more aggressive since only partial anconeus elevation is needed. The patients analyzed in the present case series expressed satisfaction with the procedure, without any complaint about symptomatic hardware or aesthetic concerns. In conclusion, this new approach to IJS application represents a valid alternative, especially for skinny patients or athletes with a bulky anconeus.

## Figures and Tables

**Table 1:** Patients’ data (acute setting).

Patients’ Data					Injury		Surgery	
Patient No.	Sex	Age (y)	Work Activity	Side Involved (Dominant Side)	Diagnosis	Acute/Chronic	Time from Injury	Treatment
**1**	M	50	MW	L (R)	Terrible triad	Acute	4 days	Coronoid and radial head fixation and LUCL repair
**2**	M	39	MW	R (R)	Terrible triad	Acute	7 days	Radial head fixation and LCL repair
**3**	M	52	MW	R (R)	PMRI and coronoid fracture	Acute	8 days	Coronoid fixation and LUCL repair
**4**	M	31	MW	R (R)	Simple dislocation	Acute	10 days	Ligaments repair
**5**	M	42	SW	R (L)	Terrible triad	Acute	12 days	LUCL repair and radial head replacement

Notes: Work activity: MW, manual worker; SW, sedentary. Side involved (dominant side): L, left; R, right.

**Table 2:** Patients’ data (chronic setting).

Patients’ Data					Injury		Surgery	
Patient No.	Sex	Age (y)	Work Activity	Side Involved (Dominant Side)	Diagnosis	Acute/Chronic	Time from Injury	Treatment
**1**	M	60	SW	R (R)	Stiffness	Chronic	5 months	Debridement arthroplasty
**2**	M	43	MW	L (R)	Stiffness with radial head resected	Chronic	12 months	Debridement and ligament reconstruction
**3**	F	54	MW	R (R)	Stiffness and instability	Chronic	10 months	Debridement
**4**	M	49	MW	R (R)	Stiffness following PLRI	Chronic	3 months	Debridement and ligament reconstruction
**5**	F	22	SW	R (L)	Stiffness	Chronic	8 months	Debridement and ligament reconstruction
**6**	M	50	MW	R (R)	Stiff with radial head prosthesis	Chronic	12 months	Debridement
**7**	M	46	MW	R (R)	stiffness and instability	Chronic	4 months	Debridement arthroplasty and LUCL reconstruction
**8**	F	58	MW	L (R)	Stiffness following radial head fracture and LUCL lesion	Chronic	7 months	Radial head prosthesis
**9**	M	21	MW	R (R)	Stiffness	Chronic	6 months	Debridement and LUCL reconstruction
**11**	F	52	SW	R (R)	Stiffness and instability	Chronic	12 months	Debridement and ligaments reconstruction
**12**	F	56	SW	R (R)	Stiffness with radial head prosthesis	Chronic	5 months	Debridement and ligaments reconstruction

Notes: Work activity: MW, manual worker; SW, sedentary. Side involved (dominant side): L, left; R, right.

**Table 3:** Outcomes and complications (acute setting).

Patients’ Data	Outcomes												Complications and Adverse Events	
Patient No.	Follow-Up (mo)	IJS Removal Time (mo)	Recurrent or Residual Instability	Patient’s Satisfaction	Flexion–Extension Arc (Degrees)		Prono-Supination Arc (Degrees)		Main Functional Outcome Score				Implant-Related (Hardware Failure, Sterile Inflammatory Reaction, Central Pin Loosening, etc.)	Surgery-Related (Heterotopic Ossification, Ulnar Neuropathy, Maluonion, Nonunion, Infection, Radioulnar Synostosis, etc.)
									MEPS		DASH			
					Pre	Post	Pre	Post	Pre	Post	Pre	Post		
**1**	12	8	No	Yes	n.e.	10–140	n.e.	80–80		90		9.1	No	No
**2**	12	8	No	Yes	n.e.	20–130	n.e.	60–80		85		9.1	No	Heterotopic ossification
**3**	12	10	No	Yes	n.e.	10–130	n.e.	80–80		95		2.3	No	No
**4**	12	6	No	Yes	n.e.	0–140	n.e.	80–80		100		4.5	Central pin loosening	No
**5**	13	5	No	Yes	n.e.	20–130	n.e.	60–50		85		9.1	Central pin loosening	No

Notes: n.e., not evaluated.

**Table 4:** Outcomes and complications (chronic setting).

Patients’ Data	Outcomes												Complications and Adverse Events	
Patient No.	Follow-Up (mo)	IJS Removal Time (mo)	Recurrent or Residual Instability	Patient’s Satisfaction	Flexion–Extension Arc (Degrees)		Prono-Supination Arc (Degrees)		Main Functional Outcome Score				Implant-Related (Hardware Failure, Sterile Inflammatory Reaction, Central Pin Loosening, etc.)	Surgery-Related (Heterotopic Ossification, Ulnar Neuropathy, Maluonion, Nonunion, Infection, Radioulnar Synostosis, etc.)
									MEPS		DASH			
					Pre	Post	Pre	Post	Pre	Post	Pre	Post		
**1**	12	4	No	Yes	20–100	10–120	80–80	80–60	65	85	25	6.8	No	Heterotopic ossification
**2**	13	6	No	Yes	40–100	20–110	40–80	60–70	55	70	38.5	15.9	Failure of hinge	Heterotopic ossification
**3**	14	8	No	Yes	30–110	20–140	50–60	80–80	60	85	22.7	11.4	Failure of hinge	No
**4**	16	13	No	Yes	30–100	10–140	80–80	80–80	60	90	22.7	9.1	Central pin loosening	No
**5**	16	5	No	Yes	20–120	10–140	80–80	80–80	80	95	20.5	2.3	No	No
**6**	18	12	No	Yes	40–90	30–130	20–30	60–60	35	85	43.2	6.8	Failure of hinge	Heterotopic ossification
**7**	18	6	No	Yes	30–110	0–140	80–80	80–80	55	100	22.7	2.3	No	No
**8**	20	6	No	Yes	30–120	20–140	50–40	80–80	55	85	38.6	4.5	No	Heterotopic ossification
**9**	20	5	No	Yes	10–100	0–140	80–60	80–80	75	100	20.5	4.5	Central pin loosening	No
**10**	21	8	No	Yes	10–90	0–140	80–60	80–80	55	100	38.5	2.3	No	No
**11**	22		No	Yes	40–110	0–140	80–80	80–80	45	95	47.7	2.3	No	No

## Data Availability

Dataset available on request from the authors.
